# China’s Non-CO_2_ Greenhouse Gas Emissions: Future Trajectories and Mitigation Options and Potential

**DOI:** 10.1038/s41598-019-52653-0

**Published:** 2019-11-06

**Authors:** Jiang Lin, Nina Khanna, Xu Liu, Fei Teng, Xin Wang

**Affiliations:** 1 Energy Analysis and Environmental Impacts Division, Energy Technologies Area, Lawrence Berkeley National Laboratory,1 Cyclotron Road, Berkeley, California, USA; 2 Department of Agricultural and Resource Economics, University of California, Berkeley, California USA; 3 Institute of Energy, Environment and Economy, Tsinghua University, Beijing, China

**Keywords:** Projection and prediction, Climate-change mitigation

## Abstract

Forecasts indicate that China’s non-carbon dioxide (CO_2_) greenhouse gas (GHG) emissions will increase rapidly from the 2014 baseline of 2 billion metric tons of CO_2_ equivalent (CO_2_e). Previous studies of the potential for mitigating non-CO_2_ GHG emissions in China have focused on timeframes through only 2030, or only on certain sectors or gases. This study uses a novel bottom-up end-use model to estimate mitigation of China’s non-CO_2_ GHGs under a Mitigation Scenario whereby today’s cost-effective and technologically feasible CO_2_ and non-CO_2_ mitigation measures are deployed through 2050. The study determines that future non-CO_2_ GHG emissions are driven largely by industrial and agricultural sources and that China could reduce those emissions by 47% by 2050 while enabling total GHG emissions to peak by 2023. Except for F-gas mitigation, few national or sectoral policies have focused on reducing non-CO_2_ GHGs. Policy, market, and other institutional support are needed to realize the cost-effective mitigation potentials identified in this study.

## Introduction

China is already the world’s largest energy consumer and largest emitter of energy-related carbon dioxide (CO_2_)^1,2^, and is also the world’s largest emitter of non-CO_2_ greenhouse gases (GHGs) emissions (excluding those related to land-use changes and forestry)^3^. Key non-CO_2_ GHGs include methane (CH_4_), nitrous oxide (N_2_O), hydrofluorocarbons (HFCs), perfluorocarbons (PFCs), sulfur hexafluoride (SF_6_), and nitrogen trifluoride (NF_3_)^4^. In 2014, China’s non-CO_2_ GHG emissions totaled 2.0 billion metric tons (Bt) of CO_2_ equivalent (CO_2_e), which exceeded the national GHG emissions from countries other than the United States, India, and Russia^4,5^. In addition, China’s emissions of non-CO_2_ GHGs are expected to increase rapidly in response to urbanization, consumer preferences, and end-user behavior^3,6^. A clear understanding of drivers and the potential for slowing the growth in energy- and non-energy-related non-CO_2_ GHGs is critical if China is to establish effective climate policies and programs that address all GHGs and enable realization of the Paris Agreement goals^7^.

Recent studies emphasize the importance of reducing short-lived climate pollutants (including non-CO_2_ GHGs such as methane and some F-gases) —in addition to significantly reducing CO_2_ emissions—in order to achieve the overall goal of limiting global temperature increase to less than 2 °C^8,9^. Shindell *et al*. and Xu *et al*. project that reducing short-lived climate pollutants could help avoid 0.6 °C in temperature increase by 2050 and halve the rate of global warming^10,11^. The recent Intergovernmental Panel on Climate Change (IPCC) report also points out the importance of concurrent deep reductions in non-CO_2_ GHG emissions, especially methane, along with net zero CO_2_ emissions to limiting global warming to 1.5 °C^12^. Despite the importance of short-lived climate pollutants, Ross *et al*. found that actions to mitigate those pollutants are often underrepresented in the nationally determined contributions (NDCs) submitted in support of the Paris Agreement and in efforts to reach China’s sustainable development goals. For instance, China’s NDCs for non-CO_2_ GHGs^13^ lack specific reduction targets.

Previous research has identified significant potential for reducing non-CO_2_ GHGs in China, but large uncertainties remain. Yao *et al*. (2016) identified potential for an approximately 30% reduction in projected non-CO_2_ GHGs by 2030^6^. Larger uncertainties surround non-CO_2_ GHG sources than pertain to CO_2_ emissions because non-CO_2_ GHG sources are contingent on production technologies^14^ and the efficacy and applicability of mitigation measures^3^. In China, data challenges and lack of awareness have hampered research on the growth and mitigation of non-CO_2_ GHG emissions, adding to the uncertainties. Wang *et al*. (2017) found that the uncertainties in China’s inventories of non-CO_2_ GHG range from a low of ±15% to a high of ±55%, with the greatest uncertainty related to N_2_O and CH_4_ emissions^15^. In addition, most previous studies have focused on one or two gases without attempting to quantify the total contribution or mitigation potential of all major non-CO_2_ GHGs. Some studies have projected China’s non-CO_2_ GHGs either to only 2020 or 2030^3,6,16,17^ or have focused only on certain gases, such as F-gases^18,19^. Other Chinese studies have evaluated the potential for deploying options for mitigating non-CO_2_ emissions in certain sectors, such as coal mining, industrial processes, and waste and wastewater, without considering other key sectors such as oil and gas or agriculture and/or without evaluating the combined effects of deploying mitigation options in various sectors^16,20^.

Building on the existing body of research, this study seeks to use a comprehensive bottom-up end-use modeling approach to understand the activity and technology drivers of non-CO_2_ GHG emissions in China through 2050 to produce a detailed outlook of possible future emission trajectories. It focuses on evaluating selected non-CO_2_ GHG mitigation options that are already considered cost-effective today in terms of total deployment potential and emission reduction efficiency, in order to quantify the existing cost-effective potential for reducing China’s future non-CO_2_ GHG emissions in addition to CO_2_ emissions.

This study employed a novel bottom-up end-use modeling approach to capture macroeconomic and physical drivers of both energy and non-energy demand in multiple sectors (e.g., agriculture, materials, waste generation) in China and the effects on CO_2_ and non-CO_2_ GHG emissions through 2050. Specifically, this analysis used the China 2050 Demand Resources and Energy Analysis Model (DREAM), which is based on an accounting framework for China’s energy and economic structure using the Long-Range Energy Alternatives Planning (LEAP) software platform developed by the Stockholm Environmental Institute. LEAP is a medium- to long-term integrated modeling tool that can be used to track energy consumption, production, and resource extraction in all sectors of an economy and to conduct analysis of long-term scenarios. Bottom-up LEAP-based models have been used extensively to model energy scenarios and CO_2_ emission trajectories for various regions, including the national and regional levels of China, as analyzed by Zhou *et al*. (2014), Khanna *et al*. (2016), Zhou *et al*. (2019), Lin *et al*. (2010), Yu *et al*. (2015), and Yang *et al*. (2017)^21–26^. However, LEAP-based models have been used only rarely to model non-CO_2_ GHGs^21–29^. Lin *et al*. (2018) used LEAP to model both CO_2_ and non-CO_2_ emissions for the city of Xiamen, but projected the reduction in non-CO_2_ emissions primarily based on reduced activities rather than through implementation of specific mitigation technologies or measures^30^.

This study represents one of the first comprehensive modeling studies of China’s total future non-CO_2_ GHG emissions and potential for mitigating them using a bottom-up approach. In the China 2050 DREAM, new non-energy modules are used to represent the major emitting sectors of agriculture, waste and wastewater, and industrial processes. The China 2050 DREAM modeling framework for energy and non-energy sectors is included in the Methods section. The 2050 DREAM captures adoption of end-use technologies and macroeconomic and sector-specific drivers of energy demand^23,31^. For non-CO_2_ GHGs, published statistics were used to calibrate historical activity levels to the latest year reported (i.e., 2016 or 2017). Table 1 presents the results of calibrating total non-CO_2_ GHG emissions, by gas, against China’s latest greenhouse gas inventory data for 2014, published in the *The People’s Republic of China Second Biennial Update Report on Climate Change*^5^.

**Table 1:** Comparison of GHG emissions as reported for 2014 and as calculated using the China 2050 DREAM.

		CO_2_	CH_4_	N_2_O	HFCs	PFC	SF_6_	Total
Energy Sector	2014 GHG Inventory	8,925	520	114	0	0	0	9,559
	DREAM Results	10,049	631	65	0	0	0	10,745
Industrial Processes	2014 GHG Inventory	1,330	0	96	214	16	61	1,718
	DREAM Results	0	0	97	233	20	43	393
Agriculture	2014 GHG Inventory	0	467	363	0	0	0	830
	DREAM Results	0	424	300	0	0	0	724
Waste and Wastewater	2014 GHG Inventory	20	138	37	0	0	0	195
	DREAM Results	0	155	15	0	0	0	170

Note: The China 2050 DREAM considers only energy-related CO_2_, not the process-based CO_2_ emissions reported in the national GHG inventory.

Source: [5].

For this study, two primary scenarios were developed to evaluate the potential impacts of non-CO_2_ GHG mitigation options, focusing on a selected set of specific technologies, measures, and policies across key energy and non-energy sectors. In addition, deployment potential, costs, and efficacy were evaluated. The Reference Scenario represents a counterfactual baseline scenario that reflects only current policies including already announced energy and carbon intensity reduction and non-fossil power generation capacity targets, assuming that the only additional non-CO_2_ mitigation measure will be the hydrochlorofluorocarbon (HCFC) phase-down schedule that already is underway under the Montreal Protocol. The CO_2_ Plus Non-CO_2_ GHG Mitigation Scenario includes key cost-effective and currently available technologies that could reduce non-CO_2_ GHG emissions, in addition to full adoption of today’s cost-effective efficiency and renewable technologies to reduce CO_2_ in selected energy and non-energy sectors. Mitigation measures for CO_2_ emissions include maximizing the adoption of cost-effective energy efficiency technologies and fully switching to cost-effective cleaner (e.g., natural gas) and/or renewable fuels by 2050. Previous analyses have focused on evaluating the cost-effective CO_2_ mitigation potential for China^22,23^, whereas this study focuses specifically on cost-effective mitigation measures for non-CO_2_ GHG emissions from various sectors that include the following:

- Coal Mining: methane oxidation and methane recovery from ventilation air
- Natural Gas: green completions; plunger lift systems; leak monitoring and repair; and low-bleed, no-bleed, or air pneumatic controllers
- Landfill: methane collection, flaring, and recovery of landfill gas for energy use
- Agriculture: humid and intermittent irrigation, improving livestock productivity, manure composting, and reducing nitrogen fertilization
- HCFC-22 Production: thermal oxidation
- Room and Mobile Air Conditioners: replacing refrigerant having high global warming potential (GWP) with low-GWP refrigerants
- Commercial Air Conditioners, Commercial and Industrial Refrigeration: improved leakage control
- Aluminum Production: mitigation of the anode effect and automated controls in the electrolytic process
- Power systems: SF_6_ recycling, leak detection and repair, equipment refurbishment, and improved SF_6_ handling

The assumptions underlying the non-CO_2_ mitigation measures and their adoption rates are presented in Supplementary Table S3 in the online Supplementary Information. More details on the overall modeling methodology and specific scenario assumptions for non-CO_2_ GHGs are provided in the Methods section and the Supplementary Information.

## Results

### Total GHG and Non-CO_2_ GHG Results

**Figure 1:**
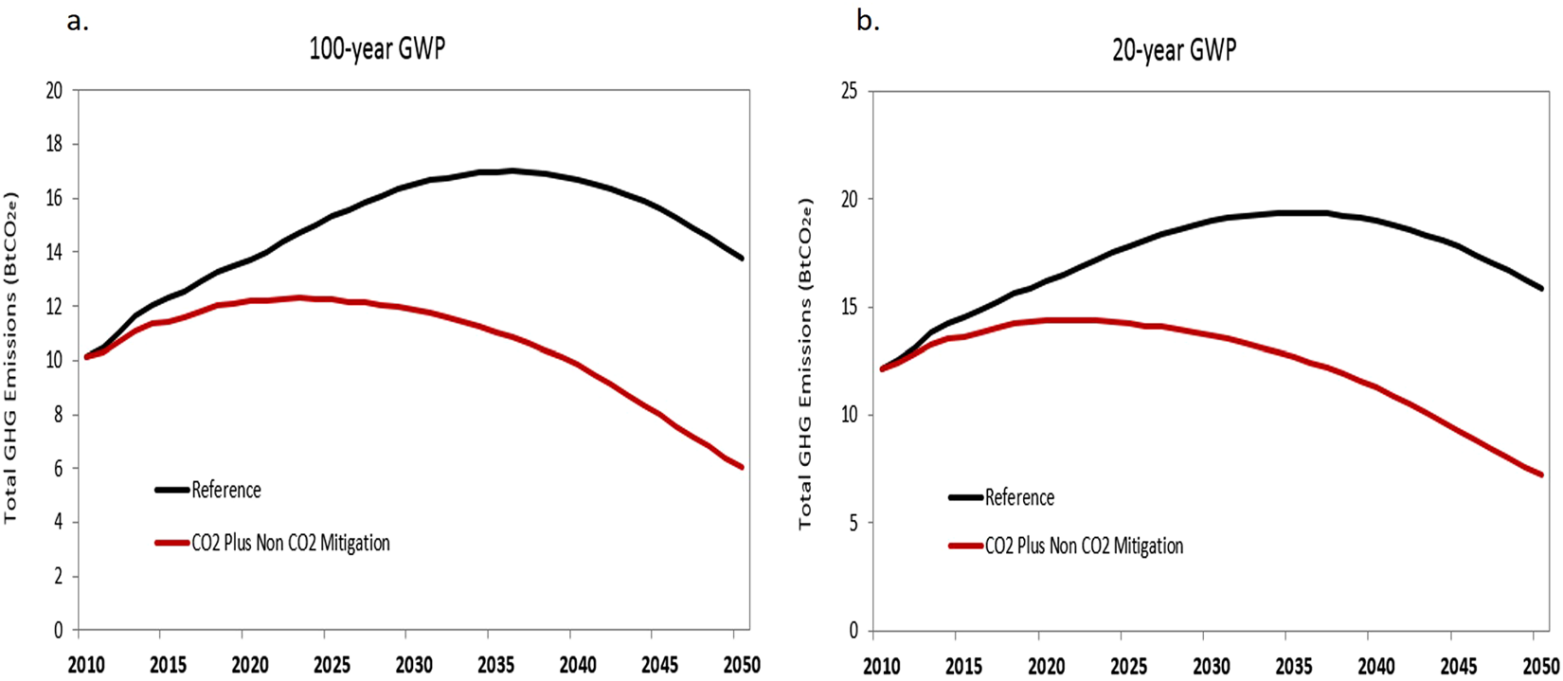
Total CO_2_ and non-CO_2_ GHG emissions by scenario from 2010 to 2050. (**a**) Total GHG emissions calculated using 100-year GWP. (**b**) Total GHG emissions calculated using 20-year GWP.

This study shows that adopting today’s cost-effective CO_2_ and non-CO_2_ GHG mitigation measures will enable China’s total GHG emissions to peak earlier at a lesser amount. The CO_2_ Plus Non-CO_2_ GHG Mitigation Scenario peaks at 12.3 Bt CO_2_e in 2023, whereas in the Reference Scenario total GHG emissions peak at 17.0 Bt CO_2_e in 2036 (using a 100-year GWP) (Fig. 1a). (Unless stated otherwise, results for the 100-year GWP are based on the *2006 IPCC Guidelines for Global Warming Potentials*, which is the most widely adopted GHG reporting convention in the United States). The GHG reductions achieved by applying non-CO_2_ GHG mitigation measures are amplified using a 20-year GWP. Under the CO_2_ Plus Non-CO_2_ Mitigation Scenario, total GHG emissions peak at 14.4 BtCO_2_e in 2020, a 25% reduction compared to the peak of 19.4 BtCO_2_e in 2035 under the Reference Scenario (Fig. 1b).

**Figure 2:**
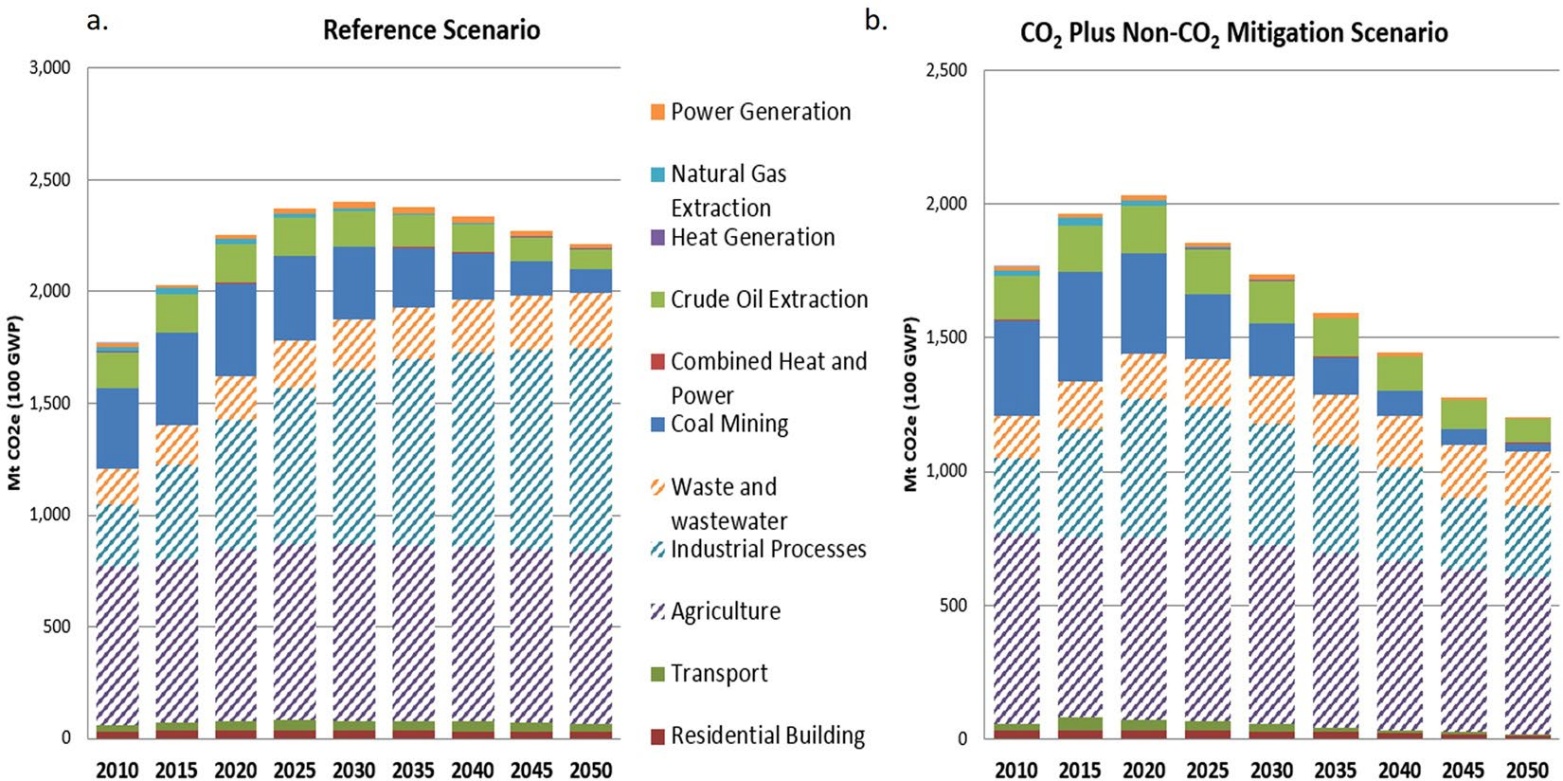
Increase in non-CO_2_ GHG emissions by sector under (**a**) Reference Scenario comprising only current policies without any non-CO_2_ GHG mitigation measures, and (**b**) CO_2_ Plus Non-CO_2_ Mitigation Scenario incorporating cost-effective mitigation measures. Patterned bars represent non-energy sectors; solid bars represent energy sectors.

Under the Reference Scenario, total non-CO_2_ GHG emissions peak in 2030 with 2400 MtCO_2_e (at 100-year GWP) (Fig. 2a). In the absence of mitigation options, non-energy sectors (represented by the patterned bars in Fig. 2a) are expected to contribute the most to future increases in China’s non-CO_2_ GHG emissions; agriculture and non-energy industrial processes each contribute more than one-third of total non-CO_2_ GHG emissions by 2050. Non-CO_2_ GHG emissions from industrial processes increase rapidly, more than tripling between 2010 and 2050 because of continued demand for industrial products such as aluminum, air conditioners, and chemical feedstock for synthetic polymers. In contrast, total non-CO_2_ GHG emissions from coal mining decrease over time, as coal use declines to meet China’s climate change commitments and to address domestic environmental and air quality concerns.

The combined mitigation measures under the CO_2_ Plus Non-CO_2_ Mitigation Scenario reduce non-CO_2_ GHGs from the 2020 peak level of 2.0 Bt CO_2_e to 1.2 Bt CO_2_e in 2050 (Fig. 2b). Compared to the Reference Scenario, the CO_2_ Plus Non-CO_2_ Mitigation Scenario would enable China to reduce future non-CO_2_ GHG emissions by 47% in 2050.

**Figure 3:**
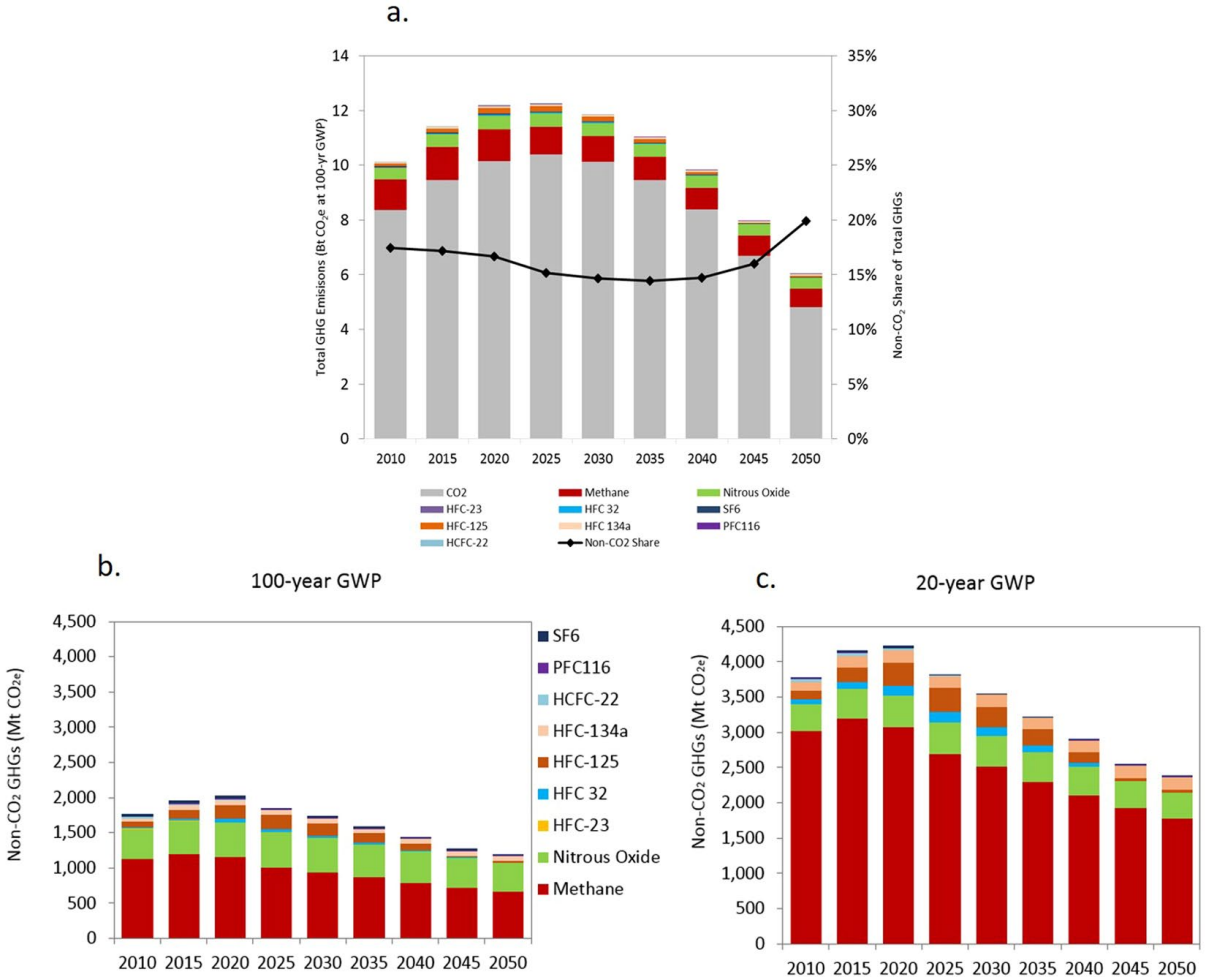
Total GHG emissions by gas under the CO_2_ Plus Non-CO_2_ Mitigation Scenario. (**a**) Total CO_2_ and non-CO_2_ emissions, (**b**) only non-CO_2_ GHG emissions using 100-year GWP, (**c**) only non-CO_2_ GHG emissions using 20-year GWP.

The percentage of non-CO_2_ emissions out of total GHGs will increase slightly from 17% in 2010 to 20% in 2050 after decreasing from the mid-2020s through 2040 (Fig. 3a). Under the CO_2_ Plus Non-CO_2_ Mitigation Scenario and using a 100-year GWP, methane and N_2_O dominate non-CO_2_ GHG emissions, accounting for 55% and 34%, respectively, in 2050 (Fig. 3b). This result suggests that cost-effective measures for mitigating CO_2_ are more readily available and can be more aggressively deployed than for non-CO_2_ emissions, leading to a significant drop in both total CO_2_ and total GHG emissions. Non-CO_2_ measures, particularly cost-effective non-CO_2_ measures, are less readily available and may be more difficult to deploy at a larger scale, such as in the agricultural sector. Agricultural policies and awareness programs are needed to change current farming and irrigation practices, which are based solely on maximizing agricultural yield.

Using a 20-year GWP that emphasizes the shorter lifetime of GHGs such as methane, the proportion of non-CO_2_ out of total GHG emissions decreases from 30% in 2015 to 24% around 2035, but then increases to 31% in 2050. Compared with using a 100-year GWP, the total annual non-CO_2_ GHG emissions almost double using a 20-year GWP (Fig. 3c). This result highlights the high GWP and relatively short lifetime of methane under the 20-year GWP accounting method. If the shorter lifetime of methane is considered through the use of a 20-year GWP, then by 2050 methane’s contribution to non-CO_2_ GHG emissions increases to 74%, whereas the proportion of N_2_O drops to 15% by 2050 (Fig. 3c). Strategies and policies that promote methane mitigation measures will have the greatest near-term effect on mitigating climate change effects.

### Non-CO_2_ GHG mitigation potential

**Figure 4:**
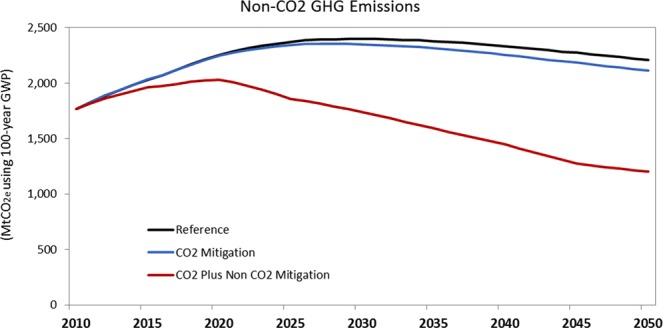
Non-CO_2_ GHG Emissions by Mitigation Scenario.

**Figure 5:**
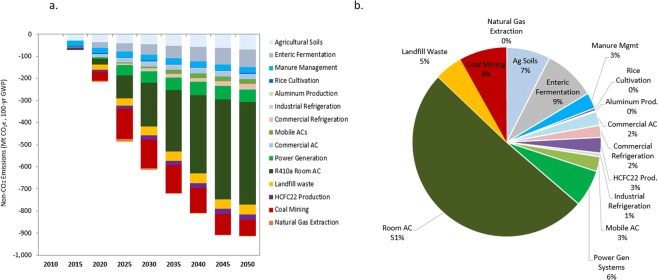
Non-CO_2_ GHG emissions reduction potential by non-energy subsector. (**a**) Total reduction potential by subsector from 2010 to 2050, calculated by comparing CO_2_ Plus Non-CO_2_ Mitigation Scenario with Reference Scenario and using 100-year GWP (**b**) subsectoral percentages of potential for reducing non-CO_2_ GHG emissions by 2050.

By 2050, China could reduce annual non-CO_2_ GHG emissions by 870 Mt CO_2_e by utilizing a combination of CO_2_ and non-CO_2_ mitigation measures (Fig. 4). CO_2_ mitigation measures such as improvements in energy efficiency, fuel switching, and lowered production activity provide only 8% of the reduction potential. The rest of the reduction potential can be achieved by implementing current cost-effective non-CO_2_ GHG mitigation measures. The adoption of those mitigation measures can help offset the expected growth in non-CO_2_ GHGs, particularly from the agricultural and industrial processes that in 2050 will produce more than 70% of non-CO_2_ GHGs (Fig. 5a).

Industrial processes such as air conditioners, HCFC-22 production, and mobile air conditioners present the greatest opportunity for reducing non-CO_2_ GHGs (Fig. 5b); activity drivers for F-gas emissions are expected to increase. By 2050, thermal oxidation during HCFC-22 production and replacing high-GWP refrigerants with low-GWP refrigerants in mobile and room air conditioners together can eliminate 510 Mt CO_2_e, or the equivalent of 90% of the total potential for reducing non-CO_2_ GHG emissions from industrial processes. The HFC phase-down schedule under the Kigali Amendment to the Montreal Protocol and the financial incentives for low-GWP refrigerant air conditioners provide an effective policy framework for reducing HFCs. Specific policies and supporting measures targeting other GHGs and industrial processes are still needed, however.

The agriculture and coal mining sectors hold a relatively large potential for emissions reduction. The agricultural sector also holds potential for large absolute reductions of N_2_O and methane emissions, but the reduction potential is a small percentage of total agricultural non-CO_2_ emissions (e.g., 23% in 2050), indicating a need for exploring other mitigation measures. Currently the greatest cost-effective reduction potential in the agricultural sector is related to livestock management, including improved manure management and enteric fermentation through improving livestock productivity, use of appropriate feed, and the conversion of manure to compost. But, unlike for other sectors, there are limited technological measures or direct strategies for reducing non-CO_2_ GHG emissions from agriculture, making it difficult to reduce agricultural non-CO_2_ emissions.

Mitigating emissions from coal mining contributes to the relatively large (70%) reduction in methane emitted by 2050. The absolute reduction in coal mining methane is greater in earlier years, because future coal production will decline to meet current energy and climate policy targets.

The waste and wastewater sectors offer additional potential for reducing methane and N_2_O emissions: by 2050 total methane emissions from those sectors can be reduced by 20%. The model accounts for mitigation measures only for landfill waste, because there currently are few cost-effective mitigation measures for the wastewater sector. The need to reduce non-CO_2_ emissions from municipal wastewater disposal and solid waste treatment is now recognized in policies such as the 13^*th*^ *Five Year Plan*, but more specific policy support is needed.

There have been few national or sectoral policies focused on reducing non-CO_2_ GHG emissions other than F-gas emissions. Policy, market, and other institutional support will be needed to realize the cost-effective mitigation potential identified in this study.

## Discussion and Policy Implications

Although the national 13^*th*^ *Five Year Plan* (*FYP*) and 13^*th*^ *Five Year GHG Emission Control Workplan for 2016 to 2020* stated that China will control non-CO_2_ GHG emissions, the plans offered no specific targets or timelines beyond a commitment under the Kigali Amendment of the Montreal Protocol^32,33^. This study identified a potential for cost-effectively mitigating 47% of non-CO_2_ GHGs by 2050 if cost-effective non-CO_2_ GHG mitigation measures are adopted in addition to CO_2_ mitigation measures. In this case, total GHG emissions peak in 2023, two years earlier than when adopting only CO_2_ mitigation measures. China has an opportunity to continue to demonstrate its global climate leadership by establishing concrete policy targets and mitigating measures for non-CO_2_ GHGs in its 14^*th*^ *FYP* and for enhanced NDCs by 2020 under the Paris Agreement framework.

The industrial sector offers the greatest potential for reducing non-CO_2_ GHGs, mostly through the cost-effective reduction of F-gas emissions in industrial processes. This reduction is partly due to the success of the Montreal Protocol, including the recent adoption of the Kigali Amendment, which sets a specific phase-down schedule for the production and consumption of key HFCs. China has also been working bilaterally and multilaterally with the United States and the European Union to phase down HFCs, and from 2014 to 2019 provided financial incentives for mitigating HFC 23 emissions. These efforts suggest that a well-established policy framework having specific reduction targets and pathways is instrumental in achieving emissions reduction potentials. However, non-CO_2_ GHG mitigation measures for other industrial processes have been promoted only generally through national policies such as the Circular Economy Promotion Law. Sector-specific policies and measures are still lacking.

Beyond sector-specific and gas-specific policies, co-control or complementary policies, such as reducing coal consumption and improving energy efficiency, can be even more effective in reducing GHG emissions. Shah *et al*. found that increasing the energy efficiency of room air conditioners by 30% through the refrigerant transition can double potential GHG reduction compared with simply switching to low-GWP refrigerants^34^.

Policies to promote reduction of methane emissions in the coal mining and waste sectors, which involve participation from diverse stakeholders and large numbers of companies, often evolve from voluntary practices to regulated standards. In the United States, for example, mitigation of methane emissions in the oil and gas sector started with public-private partnerships whereby companies voluntarily adopted methane reduction technologies in four oil and gas subsectors. That partnership then developed into a specific program, the Natural Gas STAR Methane Challenge Program, which provides flexible performance-based mechanisms beyond technology-only recommendations for companies to meet emission reduction targets. Building on the lessons learned from that voluntary program, national and state-level methane reduction targets, along with performance and technology standards, were developed to further reduce methane emissions in the oil and gas sector^35^.

China has used local pilot studies to perform policy experimentation on many issues, most recently including emissions trading schemes, and could adopt similar approaches to establish national or subnational targets for reducing non-CO_2_ GHG emissions. Such an experimental approach can help target the application of mitigation technologies in selected sectors or regions and identify best practices that can then be scaled up.

Mitigation of methane and N_2_O emissions in the agricultural sector may be the greatest challenge, because many mitigation options require the participation of millions of farmers in a highly decentralized sector. The choice of options also needs to account for impacts on yields and material inputs. As a result, robust institutional mechanisms are needed to disseminate cost-effective mitigation options while promoting behavioral changes to traditional farming practices. There is little international experience with mitigating non-CO_2_ GHG emissions in the agricultural sector. More research is needed to fully capture the mitigation potential in the agricultural sector.

## Conclusions

This study describes one possible pathway for China to reduce its total GHG emissions, which is by expanding the adoption of both CO_2_ and non-CO_2_ mitigation measures that are cost-effective now. This study does not attempt to project what will happen in China, given the long time frame (to 2050) and inherent uncertainties surrounding emissions factors, costs trends, pace of technological improvements, and rates of market adoption. Rather, this study is meant to be a first, comprehensive bottom-up assessment of the potential for cost-effectively reducing non-CO_2_ GHG emissions in China under a possible scenario. The study incorporates the presumption that deployment of commercially available mitigation measures can be increased via policy, market, and other institutional supports.

This study utilizes average emissions factors for many sources of methane and N_2_O emissions, although a wide range of emissions factors are presented in recent inventory guidelines and reports. In addition, simplifying assumptions are made about leakage rates for HFC—no attempt was made to quantify emissions from the various stages in the life-cycle of air conditioning equipment. Compared to other sectoral reports, relatively conservative assumptions were made about the adoption of non-CO_2_ mitigation measures in several subsectors, especially as regards to replacing high-GWP refrigerants with low-GWP alternatives for mobile and room air conditioners. For other sectors, however, such as the landfill sector, predictions of the market penetration of mitigation measures are higher in this study than in many other studies. All of these variables introduce uncertainties in the estimates of future non-CO_2_ emissions, but the uncertainties do not significantly change the observed trends and the key findings of this analysis.

To better understand how much China can reduce its non-CO_2_ GHG emissions in the short and mid-terms, more in-depth assessments of the cost-effectiveness must be conducted using China-specific costs and market conditions. Doing so will further the applicability of mitigation measures specifically to China. In addition, the scope of non-CO_2_ mitigation measures must be expanded to other subsectors, including non-conventional gas extraction. Interactions between energy systems and non-energy systems, as well as behavioral changes in meat consumption and waste management, could also be considered further in the model, along with more detailed sensitivity analysis of scenarios. To achieve the mitigation potential that this analysis has identified, concrete policy recommendations and strategies for sector-specific measures must be developed in collaboration with Chinese stakeholders.

## Methods

### Modeling framework

This analysis utilized the China 2050 Demand Resources and Energy Analysis Model (DREAM), which is based on an accounting framework of China’s energy and economic structure using the Long-Range Energy Alternatives Planning (LEAP) software platform developed by the Stockholm Environmental Institute. Previously published reports and papers^21,23,29,36^ have presented in-depth discussion of the overall modeling methodology, key sectoral drivers, and modeling parameters, as well as the assumptions and basis for future projections of energy demand drivers. The validity of the chosen model and modeling applications has been examined by others, including Li and Qi^37^ and Bellevrat^38^. Zheng *et al*. compared the underlying assumptions and modeling approaches of the China 2050 DREAM model with other bottom-up energy and emissions models for China, finding that results from DREAM reference scenarios were within the range of contemporaneous published energy and emissions outlooks for China^39^.

The China 2050 DREAM model includes a demand module consisting of five energy demand subsectors (residential buildings, commercial buildings, industry, transport, and agriculture) and energy transformation modules consisting of energy production, transmission, and distribution subsectors. The modules represent energy extraction and transformation sectors, such as the energy required to extract fossil fuels, and a power sector based on specialized generation dispatch algorithms. Using LEAP, the model captures adoption of end-use technologies as well as macroeconomic and sector-specific drivers of energy demand. The model enables detailed consideration of technological development—industrial production, equipment efficiency, residential appliance usage, vehicle ownership, power sector efficiency, lighting, and heating usage—as a way to evaluate China’s path toward reducing energy and emissions at each subsector’s end-use level. Published energy sector statistics were used to prepare a time-series database representing primary energy use that accounts for supply-side losses. After the model was built from the bottom up, sector- and fuel-specific consumption data were calibrated by comparing the calculated results with the end-use energy data reported for the most recent year.

**Figure 6:**
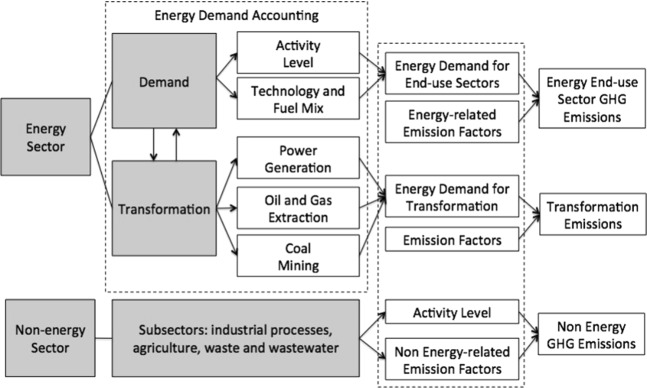
China 2050 DREAM modeling framework for energy and non-energy sectors.

Figure 6 shows the China 2050 DREAM modeling framework applied to energy and non-energy sectors.

Key drivers of future energy use in the model include activity drivers (total population growth, urbanization, building and vehicle stock, commodity production); economic drivers (total GDP, value-added GDP, income); trends in energy intensity (for energy-using equipment and appliances); and trends in carbon intensity. These factors are, in turn, driven by changes in consumer preferences, settlement/population and infrastructure patterns, technical changes, and overall economic conditions. Key macroeconomic parameters such as economic growth, population, and urbanization are aligned with international sources (e.g., the United Nations World Population Prospects^40^) as well as Chinese sources (e.g., China Energy Research Institute reports^41^).

More specifically, urbanization plus growth in household income drive energy consumption in residential building, because urban households generally consume more commercially supplied energy than do rural households and because rising household incomes correspond to increased housing unit size (and thus increased heating, cooling, and lighting loads), along with appliance ownership. Energy demand for commercial buildings is driven by two similar factors: building area (floor space) and end-use intensity related to heating, cooling, and lighting (megajoules per m^2^). For the industrial sector, the model includes 12 energy-intensive industries that are driven by key physical drivers related to expanding demand for the built environment (e.g., for housing, agricultural products, and plastics), and 18 light manufacturing industries that deliver value-added products. Transportation demand is driven by trends in freight and passenger transport. Freight transport is calculated as a function of economic activity (measured by GDP); passenger transport is based on average number of vehicle-kilometers traveled by each mode of transportation (bus, train, or car).

The energy transformation module in the China 2050 DREAM accounts for the energy required to extract primary fossil fuels and to operate processing and conversion plants that produce derived products such as electricity, coke, and petroleum products (Fig. 6). The energy extraction and processing subsectors in the model include coal mining, oil extraction, natural gas extraction, coking, oil refining, and coal liquefaction. Technological improvements, resource quality, and resource limits are considered when accounting for the energy required to extract, process, and convert these sources of energy.

To capture the non-CO_2_ GHG emissions from key non-energy-consuming activities, a non-energy module was added to the China 2050 DREAM to represent the major emitting sectors of agriculture, waste and wastewater, and industrial processes (Fig. 6). Non-energy-consuming activities in these sectors, such as rice cultivation, livestock management, wastewater treatment, and industrial processes, are documented sources of non-CO_2_ GHG emissions, with increased activity expected in some sectors as China continues its economic transition. The drivers of these non-energy sectoral activities and the related non-CO_2_ GHG emissions are discussed in greater detail in the section below, titled Scenario Analysis. Future projections of these drivers including key data points are detailed in the online Supplementary Information. For the energy sector, published statistics were used to calibrate historical activity levels, and total emissions were calibrated against the latest published data (collected in 2014^5^). The model captures cross-sectoral linkages between energy activities (e.g., electricity demand) and non-energy activities (e.g., power generation, system production).

## Scenario Analysis

Two primary scenarios were developed for this analysis: a reference or baseline scenario and a Non-CO_2_ GHG Mitigation Scenario. The scenarios evaluate potential trajectories for China’s GHG emissions from 2015 through 2050. A scenario focusing only on CO_2_ mitigation, developed in a previous study, was also referenced to compare the accompanying benefits to reducing non-CO_2_ GHG emissions from CO_2_ mitigation measures such as efficiency and fuel switching^23,42^. The three scenarios are described below.

1. **Reference Scenario:** assumes all energy-related policies currently in place, along with autonomous technological improvements, will continue to affect all energy demand, supply, and transformation. The reference, or baseline, scenario assumes that no new policies will be developed. This scenario also assumes that no non-CO_2_ mitigation measures will be implemented before 2050, exception for efforts made to reach the Montreal Protocol targets for HFCs from HCFC-22 production.
2. **CO**_**2**_ **Mitigation Scenario**: assumes that the greatest efficiency and renewable achievements that currently are cost effective or nearly cost effective are adopted fully in all energy demand sectors and the power sector by 2050 to reduce CO_2_ emissions.
3. **CO**_**2**_ **Plus Non-CO**_**2**_ **GHG Mitigation Scenario:** includes key commercially available technologies that currently are cost effective or nearly cost effective. This scenario includes the annualized costs of CO_2_e reductions of less than 50 RMB/tCO_2_e, with the exception of the slightly higher costs for replacing hydrofluoro-olefin (HFO)-1234yf in new light-duty vehicles^3,16^, which potentially could reduce non-CO_2_ GHG emissions in the energy and non-energy sectors. This scenario also includes mitigation measures that are beginning to be adopted in the Chinese market without reference to specific policies, assuming that those mitigation measures started being adopted only after 2010.

## Data and Assumptions

For historical data, the China 2050 DREAM inputs were calibrated with the latest reported national statistics for both energy and non-energy activity variables such as population, households, industrial production, agricultural activity, and waste generation. Energy consumption data by fuel and by sector also were calibrated to the latest published national energy balances. Calculations of emissions data used China-specific fuel energy content and emissions factors for CO_2_ and a mix of emissions intensities from the 2010 *Chinese Provincial Guidelines for GHG Inventories*, the 2006 *IPCC Guidelines for GHG Inventories* and other sector-specific Chinese studies for non-CO_2_ GHGs^14,43^. Detailed data inputs and assumptions for future activity drivers, activity levels and emission factors can be found in Supplementary Tables S1, S2, and S3.

## Supplementary information

Supplementary Information

## Data Availability

The data that support the plots within this paper and other findings of this study are available from the corresponding author upon reasonable request.
