# Supplementary material for: China’s Non-CO2 Greenhouse Gas Emissions: Future Trajectories and Mitigation Options and Potential
Source: Sci Rep. 2019 Nov 6;9:16095. doi: 10.1038/s41598-019-52653-0 (PMC6834562; doi:10.1038/s41598-019-52653-0)
Supplement: Supplementary file 1 — Supplementary Information [file 41598_2019_52653_MOESM1_ESM.docx]

**Supplementary Information**

**China’s Non-CO_2_ Greenhouse Gas Emissions:
Future Trajectories and Mitigation Options and Potential**

Jiang LIN^1,2^*, Nina KHANNA^1^, Xu LIU^1^, Fei TENG^3,^*, Xin WANG^3^

Energy Analysis and Environmental Impacts Division. Energy Technologies Area. Lawrence Berkeley National Laboratory. 1 Cyclotron Road, Berkeley, California, USA.

^2^ Department of Agricultural and Resource Economics, University of California, Berkeley, California, USA.

3 Institute of Energy, Environment and Economy. Tsinghua University. Beijing, China.

* Corresponding authors: Jiang LIN, Energy Analysis and Environmental Impacts Division, Lawrence Berkeley National Laboratory. One Cyclotron Road, MS90R2121, Berkeley, California, 94720. USA. E-mail: [j_lin@lbl.gov](mailto:j_lin@lbl.gov) .

Fei TENG, Institute of Energy, Environment and Economy, Tsinghua University, 1 Qinghuayuan, Haidian, Beijing 100084, People’s Republic of China, e-mail: [tengfei@tsinghua.edu.cn](mailto:tengfei@tsinghua.edu.cn)

**Key Model Inputs**

The key activity drivers and assumed GHG emissions intensities by sector in this study are listed in Table S1. Total emissions under the Reference Scenario (with no mitigation) are calculated as total activity levels (shown for selected years in Table S2) multiplied by the constant emissions factors shown in Table S1.

**Table S1. Key non-CO_2_ GHG activity drivers and assumptions regarding emission factors**

|  | **GHG Activity Driver** | **Basis for Activity Projections** | **Sources for Emission Factors (EF)** |
| --- | --- | --- | --- |
| ***Energy Sectors*** | | | |
| Coal Mining: CH_4_ | Coal production, underground and surface mining | Endogenously calculated in model based on demand. | [43] |
| Oil and Natural Gas Sector: CH_4_ | Oil and natural gas production | Endogenously calculated in model based on demand. Production forecast based on resource curves. | Tier 1 default [14]; average values for extraction and transmission and distribution |
| Stationary Combustion: CH_4_, N_2_O | Oil and coal boilers in industrial and power sectors | Endogenously calculated in model based on industrial energy use and power generation. | Tier 1 default [14] |
| Mobile Combustion: CH_4_, N_2_O | Trucks, buses, passenger vehicles, rail, water, air | Fossil fuel consumption by vehicles based on total transport activity by model and fuel mix. | Tier 1 default [14]; average values |
| Residential Biomass: CH_4_, N_2_O | Rural biomass heaters and cookers | Heating and cooling demand calculated endogenously by model and assumed fuel mix for rural households. | Weighted by proportions of firewood and stalk [43] |
| ***Agriculture*** | | | |
| Rice Cultivation: CH_4_ | Harvest areas for four different growth seasons | Projected growth rates based on *China Agriculture Outlook* [44]. | Average EFs for each type of growth season [43] |
| Enteric Fermentation: CH_4_ | Nine primary types of livestock | Varies by animal type: growth based on population, meat consumption, and linear regressions. | Average EFs for type of livestock [43] |
| Manure Management: CH_4_, N_2_O | Nine primary types of livestock | Varies by animal type: growth based on population, meat consumption, and linear regressions. | Average EF for each type of livestock [43] |
| Agricultural Soils: N_2_O | Nitrogenous fertilizers | Calculated by model based on changes in sown area and fertilizer intensity. | Tier 1 default EFs for direct and indirect emissions [14] |
| Manure Fertilizer: N_2_O | Manure from livestock | Linked to projected quantities of livestock, with constant manure rates. | Direct [43]; indirect [14] |
| ***Waste and Wastewater*** | | | |
| Solid Waste: CH_4_ | Solid waste | Regression of landfill waste based on population and per capita GDP. | Emissions calculated using IPCC First Order Decay Model [14] |
| Domestic Wastewater: CH_4，_N_2_O | Organics in wastewater | Per capita biochemical oxygen demand using Asian regional defaults. | CH_4_: [16]  N_2_O: [14] |
| Industrial Wastewater: CH_4,_ N_2_O | Chemical oxygen demand (COD) in wastewater | Total COD and COD removed based on regression to industrial value added. |  |
| ***Industrial Processes*** | | | |
| Nitric Acid: N_2_O | Nitric acid production | Constant growth rate based on Yang et al. projections to 2020 [16]. | Production process-weighted EF [16] |
| Adipic Acid: N_2_O | Adipic acid production | Constant growth rate based on Yang et al. projections to 2020 [16]. | [43] |
| HCFC-22 Production: HFC-23 | HCFC-22 production | Feedstock use: Same growth as national GDP. Non-feedstock use: phase-down based on Montreal Protocol HCFC schedule. | [43] |
| Aluminum Production: PFC-116 | Primary aluminum production | Calculated endogenously by model based on domestic aluminum demand and assumed primary share of production. | [43] |
| Semi-conductors: PFC-116 | Given data limitations, total emissions were projected using same growth rates as Yang et al. (2014 through 2020), then slower growth was assumed based on national GDP growth rates through 2050 [16] | | |
| Mobile ACs: HFC-134a | Mobile ACs | Projected stock of trucks, buses, and passenger vehicles using lifetime of 10 years. | [18, 45] |
| Room ACs: HFC-125, HFC-32 | Room ACs | Projected stock of room air conditioners based on household ownership and lifetime of 10 years. | [46] |
| Commercial ACs: HFC-134a | Commercial Cooling Demand | Projected total cooling demand as a function of growth in commercial floorspace. | [57] |
| Commercial Refrigeration: HFC-134a, HFC-125, HFC-32 | Commercial Refrigeration | Projected total refrigeration demand as a function of value-added GDP for the tertiary sector. | [57] |
| Industrial Refrigeration: HFC-134a, HFC-125 | Industrial Refrigeration | Projected total refrigeration demand as a function of value-added GDP for the secondary sector. | [57] |
| Power Generation Systems: SF6 | Power generation Output | Calculated endogenously by model. | [16] |

The specific assumed future activity levels for non-CO_2_ GHG activity drivers are shown in Table S2 for key milestone years of 2015, 2030, and 2050.

**Table S2. Non-CO_2_ GHG Emissions Activity Drivers and Projections for Key Years, by Scenario**

|  |  | **Reference Scenario** | | | | **Mitigation Scenario** | | | |
| --- | --- | --- | --- | --- | --- | --- | --- | --- | --- |
|  |  | **2010** | **2015** | **2030** | **2050** | **2010** | **2015** | **2030** | **2050** |
| **Agricultural** |  |  |  |  |  |  |  |  |  |
| Livestock |  |  |  |  |  |  |  |  |  |
| Sheep and Goats | million | 281 | 311 | 393 | 375 | Same as Reference Scenario | | | |
| Hogs | million | 465 | 451 | 496 | 472 |  |  |  |  |
| Camels | million | 0 | 0 | 0 | 0 |  |  |  |  |
| Mules | million | 3 | 2 | 1 | 1 |  |  |  |  |
| Donkeys | million | 6 | 5 | 3 | 3 |  |  |  |  |
| Horses | million | 7 | 6 | 4 | 4 |  |  |  |  |
| Cattle and buffalo | million | 106 | 108 | 128 | 122 |  |  |  |  |
| Rice Cultivation |  |  |  |  |  |  |  |  |  |
| Grow Area | million ha | 32 | 32 | 31 | 31 |  |  |  |  |
| **Industrial Process** |  |  |  |  |  |  |  |  |  |
| Aluminum Production | Mt | 16 | 28 | 22 | 15 | Same as Reference Scenario | | | |
| Adipic Acid | Mt | 1 | 1 | 1 | 1 |  |  |  |  |
| Nitric Acid | Mt | 2 | 3 | 4 | 4 |  |  |  |  |
| HCFC-22 Production | kt | 21 | 29 | 52 | 72 |  |  |  |  |
| R410a Room AC | million | 25 | 41 | 105 | 122 |  |  |  |  |
| HCFC22 Room AC | million | 5 | 5 | 0 | 0 |  |  |  |  |
| **Wastewater** |  |  |  |  |  |  |  |  |  |
| Industrial Wastewater | Mt | 24 | 27 | 42 | 53 | Same as Reference Scenario | | | |
| Domestic Wastewater | kg/cap | 29 | 29 | 31 | 31 |  |  |  |  |
| **Energy** |  |  |  |  |  |  |  |  |  |
| Coal production | Mtce | 2109 | 2433 | 1909 | 611 | 2109 | 2447 | 1929 | 619 |
| Gas production | Mtce | 113 | 137 | 53 | 3 | 113 | 140 | 54 | 3 |
| Oil production | Mtce | 306 | 320 | 295 | 167 | 306 | 320 | 295 | 167 |
| Transportation final energy consumption | Mtce | 324 | 462 | 708 | 798 | 324 | 455 | 633 | 649 |
| Manufacturing |  |  |  |  |  |  |  |  |  |
| Food Beverage and Tobacco | Mtce | 44 | 61 | 105 | 91 | 44 | 50 | 83 | 107 |
| Textile and Chemical Fiber and Related Products | Mtce | 55 | 72 | 97 | 84 | 55 | 57 | 74 | 83 |
| Paper and Paper Products | Mtce | 35 | 41 | 53 | 50 | 35 | 40 | 48 | 46 |
| Chemical Materials and Products | Mtce | 311 | 384 | 507 | 428 | 311 | 378 | 412 | 341 |
| Rubber and Plastics | Mtce | 19 | 27 | 57 | 30 | 19 | 24 | 34 | 32 |
| Non Metal Mineral Products | Mtce | 284 | 352 | 319 | 223 | 284 | 333 | 255 | 152 |
| Ferrous Metals | Mtce | 559 | 667 | 707 | 525 | 559 | 629 | 532 | 265 |
| Non Ferrous Metals | Mtce | 70 | 116 | 115 | 95 | 70 | 112 | 102 | 56 |
| Metal Products | Mtce | 17 | 26 | 45 | 48 | 17 | 22 | 25 | 24 |
| Machinery | Mtce | 33 | 45 | 82 | 58 | 33 | 43 | 74 | 74 |
| Transport Equipment | Mtce | 22 | 34 | 76 | 57 | 22 | 31 | 53 | 60 |
| Electric and Electronic Equipment | Mtce | 23 | 29 | 58 | 35 | 23 | 28 | 36 | 35 |

Note: Mt is million metric tons, kt is thousand metric tons, ha is hectares, kg/cap is kilograms per capita, and Mtce is million metric tons of coal equivalent, the standard unit for energy used in Chinese statistics and equal to 29.27 million gigajoules.

**Mitigation Options**

The CO_2_ Plus Non-CO_2_ Mitigation Scenario includes key cost-effective and currently available technologies that could reduce non-CO_2_ GHG emissions in the modeled energy and non-energy sectors (Table S3). Cost-effectiveness was used as a screening criterion for identifying mitigation options to include in the scope of this study. As shown in Table A-3, the costs of the selected mitigation technologies and options identified through literature review are all less than 72 RMB/tCO_2e_, except for the higher cost of replacing HFC-134a with HFO-1234yf in new cars—a maximum of 200 RMB/ tCO_2e_. Based on their low costs for abating CO_2_e, the mitigation measures described in Table A-3 are considered to be cost effective or nearly cost effective, and their deployment is included in the Mitigation Scenario. The Mitigation Scenario also includes measures that are beginning to be adopted in the Chinese market in the absence of specific policies. The lack of data on historical adoption rates for mitigation measures required the simplifying assumption that adoption started after 2010, reaching current market levels by 2015.

**Table S3. Key assumptions for mitigation measures and adoption rates under the CO_2_ Plus Non-CO_2_ Mitigation Scenario**

| **Sector and GHG Targeted** | **Mitigation Measure** | **Details of Mitigation Measure** | **Assumptions Underlying Mitigation Adoption Rate** | **Cost Estimates or Other Cost-related Information** |
| --- | --- | --- | --- | --- |
| ***Energy Sector*** | | | |  |
| Coal Mining: CH_4_ | Ventilation air methane (VAM) oxidation and drainage and flare systems | VAM oxidation combusts low- concentration methane into H_2_O and CO_2_. Drainage and flare systems recover high-concentration methane from underground coal seam. | Assume linear growth in adoption of VAM oxidation and recovery to 42% by 2050. Assume 28% adoption of drainage and flare systems by 2025, and constant thereafter [3,16]. | 2.3-71.8 RMB/tCO_2e_ [16] |
| Oil and Natural Gas Sector: CH_4_ | Green completions, plunger lift systems, leak monitoring and repair, and low- or no-bleed or air pneumatic controllers | Green completions use portable equipment to capture methane during well completion.  Plunger lift systems lift a column of accumulated fluid out of a well to replace beam lifts^[[1]](#footnote-1)^ and well blowdowns (i.e., venting) [47].  Using low- or no-bleed (i.e., low or no venting) or air pneumatic controllers can reduce methane leakage [3, 48-49]. | Assume emissions reduction rate of each technology increases linearly to 20% of its maximum reduction rates from 2015 to 2020, then gradually reaches its maximum reduction rate by 2025 [3, 48-49]. | Low-bleed or no-bleed or air pneumatic controllers: $4.44/tCO_2e_[9];  All measures are believed to be cost-effective [49] |
| ***Agriculture*** | | | |  |
| Rice Cultivation: CH_4_ | Changing irrigation method | Examples include alternate wetting and drying. | Constant 30% reduction from the baseline emissions of rice cultivation from 2015 through 2050 (based on expert interview). | There are few studies on the cost of mitigation measures in the agricultural sector in China. However, these measures are believed to be ready to use in China with little cost, based on expert interview [44]. |
| Enteric Fermentation: CH_4_ | Improving nutritional balance of livestock feed and improving feed digestibility | Increased rate of meat and dairy production from livestock. | Assume linear growth of emission reduction rate from 10% in 2020 to 30% in 2050 (based on expert interview). |  |
| Manure Management: CH_4_, N_2_O | Converting manure to compost | Compost production, one way of managing dry manure, can achieve economical methane reductions [50]. | Assume linear growth of emission reduction rate from 0 in 2010 to 50% in 2015, remaining constant thereafter (based on expert interview). |  |
| Agricultural Soils: N_2_O | Decreasing application of nitrogen fertilizer | Reduce nitrogen fertilizer application. | Assume 20% reduction in fertilizer application intensity from 2010 to 2015, rising to 50% reduction in 2050, based on expert interviews [56]. |  |
| ***Waste*** | | | |  |
| Solid Waste: CH_4_ | Collection and flaring and capturing landfill gas for energy use | Collect and directly flare methane released from landfills or use for electricity generation. | Market penetration assumed to increase to 90% in 2050. [3] | -$2/tCO_2e_[3] |
| ***Industrial Processes*** | | | |  |
| Mobile ACs: HFC-134a | Replacing HFC-134a with HFO-1234yf | Replace HFC-134a with low-GWP alternative refrigerants such as HFO-1234yf. | Assume market penetration (in new car sales) increases linearly from 5% in 2020 to 50% in 2030 and 100% in 2035 [16]. | 104-171RMB/tCO_2e_ or an additional 500 RMB for each new vehicle [16] |
| Room ACs: HFC-125, HFC-32 | Replacing HCFC-22/HFC-410a with alternative refrigerants, particularly propane. | Replace HFC-410a with R290, an alternative low-GWP refrigerant. | Assume market penetration (of new room ACs) increases from 10% to 50% from 2020 to 2030, and reaches 100% by 2045 [16]. | 26.7 RMB/tCO_2e_[16] |
| Commercial ACs: HFC-134a | Improving leakage control | Reduce annual leakage of emissions banked in equipment in use. | Assume reduction in leakage increases from 0% in 2015 to 30% by 2025. | No cost estimates, but already being implemented in EU as part of EU F-gas regulations |
| Commercial Refrigeration: HFC-134a, HFC-125, HFC-32 | Improving leakage control | Reduces annual leakage from emissions banked in equipment in use. | Assume reduction in leakage increases from 0% in 2015 to 33% by 2030 | No cost estimates available |
| Industrial Refrigeration: HFC-134a, HFC-125 | Improved leakage control | Reduces annual leakage from emissions banked in equipment in use. | Assume reduction in leakage increases from 0% in 2015 to 42% by 2025 | No cost estimates, but already implemented in EU and other Annex I countries |
| HCFC-22 Production: HFC-23 | Thermal oxidation | Thermal oxidation decomposes HFC-23 to CO_2_ and HF using a high-temperature flame in the presence of oxygen. | Assume application rate reaches 40% in 2015, doubles in 2020, and reaches 100% in 2025 [16]. | 5.8 RMB/tCO_2e_[16] |
| Aluminum Production: PFC-116 | Automated extinguisher and automated controls for the electrolytic processes | Reduces the duration of anode effects (AED) by automatically controlling alumina production and optimizing molecular ratios to prevent release of PFC-116. | Assume market penetration of a combination of these two technologies is 30% in 2011 and increases to a maximum of 50% in 2020, remaining constant thereafter. [16]. | 4-5 RMB/tCO_2e_  [16] |
| Power Generation Systems: SF6 | SF_6_ recycling, leak detection and repair (LDAR), equipment refurbishment, and improved SF_6_ handling. | SF_6_ recycling reduces vented SF_6_ emissions; LDAR reduces periodic equipment leakage of SF_6_; equipment refurbishment reduces emissions from chronically leaking equipment; improved SF_6_ handling reduces associated leaks. | Assume market penetration of equipment refurbishment increases linearly to 4% in 2020 and a maximum of 20% by 2025. For the other three measures, assume market penetration reaches 20% in 2020 and 100% in 2025 [3]. | SF_6_ recycling: $0.45/tCO_2e_; LDAR: $1.98/tCO_2e_;  equipment refurbishment: $1.65/tCO_2e_; and improved SF_6_ handling -$1.2/tCO_2e_[3];  overall 40 RMB/tCO_2e_[6] |

For other cost-effective and available non-CO_2_ mitigation technologies and measures that have not yet been adopted, adoption is assumed to ramp up from 2015 through 2025 as a result of expected policies and targets focused on non-CO_2_ GHGs, as indicated in the *13^th^ FYP for Climate Change* [33] and the HFC phase-down targets set in the Kigali Amendment to the Montreal Protocol (in most cases). In some cases, such as in the agricultural sector, landfill waste, and use of low-GWP refrigerant in mobile and room air conditioners, assumptions regarding mitigation strategies are based on expert feedback on the current and expected future pace of deployment. In addition to market adoption rates, literature and expert interviews [3, 6, 49, 51-55] assisted the calculation of total maximum reduction rates for non-CO_2_ GHG for a given measure or technology.

**Supplementary References**

[44] China Department of Agriculture. *China Agriculture Outlook (2015-2024) (in Chinese)* (China Agricultural Science and Technology Press, Beijing, 2015).

[45] China Automotive Technology and Research Center. *HFCs Mitigation Pathway in Mobile Vehicles* *(in Chinese)* (2017).

[46] Wan, T. et al. Environmental benefits of phase-out HCFC-22 in the residential air-conditioner sector in China. *Adv Clim Chan Res* **2**, 86-92 (2011). Doi:10.3724/SP.J.1248.2011.00086

[47] United States Environmental Protection Agency. *Installing Plunger Lift Systems in Gas Wells* (2016). <https://www.epa.gov/sites/production/files/2016-06/documents/ll_plungerlift.pdf>

[48] Brink, S., Godfrey, H., Kang, M., Lyser, S., Majkut, J., Mignotte, S. *Methane Mitigation Opportunities in China* (2013). <https://www.princeton.edu/~mauzeral/teaching/WWS591e_Methane_Workshop_FinalReport%202013.pdf>.

[49] Natural Resources Defense Council*. Controlling Methane Emissions from the Natural Gas Systems* (in Chinese) (2012). <http://www.nrdc.cn/Public/uploads/2016-12-04/5843d6a697a95.pdf>

[50] Kotin, A., Noble, M., Merrill, J. *Diversified Strategies for Reducing Methane Emissions from Dairy Operations* (2015). <http://calclimateag.org/wp-content/uploads/2015/11/Diversified-Strategies-for-Methane-in-Dairies-Oct.-2015.pdf>.

[51] United Nations Framework Convention on Climate Change. *Implications of the establishment of new hydrochlorofluorocarbon-22 (HCFC-22) facilities seeking to obtain certified emission reductions for the destruction of hydrofluorocarbon-23 (HFC-23)*. (2011).

[52] Zhang, Y., Chai, R., Fu, L., Liu, L., Dong, H. Greenhouse gas emissions from major agricultural activities in China and corresponding mitigation strategies (in Chinese). *Journal of Zhejiang University (Agric. & Life Sci.)* ***38***, 97-107 (2012).

[53] Zeng, B., Zhong, T., Tan, Z. Methane emission and abatement strategy in animal husbandry (in Chinese). *Chinese* *Journal of Eco-Agriculture* **17**, 811−816 (2009)

[54] Shao, M., Sun, J., Ruan, G. Review on greenhouse gases emission and the reduction technology in rice fields (in Chinese). *Zhejiang Agriculture* **23**, 181-187 (2011).

[55] Li, S., Jin, X., Fan, X., Huang, W., Cao, Z. Ruminant production and carbon emission reduction measures (in Chinese). *Chinese Journal of Animal Nutrition* **22**, 2-9 (2010).

[56] Reay, D.S. et al. Global agriculture and nitrous oxide emissions. *Nat Clim Chang* **2**, 410-416 (2012).

[57] Purohit, P. and Hoglund-Isaksson, L. Global emissions of fluorinated greenhouse gases 2005-2050 with abatement potential and costs. *Atmos Chem Phys,* 17, 2795-2816 (2017).

1. Beam lifts are the most common method of using pumps to remove liquids from gas wells. [↑](#footnote-ref-1)
